# Supplementary material for: Decoupling gene functions from knockout effects by evolutionary analyses
Source: Natl Sci Rev. 2020 Apr 24;7(7):1169–80. doi: 10.1093/nsr/nwaa079 (PMC8288921; doi:10.1093/nsr/nwaa079)
Supplement: nwaa079_Supplemental_File [file nwaa079_supplemental_file.pdf]

Supporting Information of

"Decoupling gene functions from knockout effects by evolutionary analyses"

Li Liu[#], Mengdi Liu[#], Di Zhang, Shanjun Deng, Piaopiao Chen, Jing Yang, Yunhan Xie & Xionglei He*

State Key Laboratory of Biocontrol, School of Life Sciences, Sun Yat-sen University, Guangzhou 510275, China

This file contains:
Legends of Tables S1 to S8
Figs. S1 to S10

Legends of supplementary tables

Table S1: RNA-seq-based gene expression levels for each HAP4 deletion and wild-type strain.

Table S2: RNA-seq-based expression changes (P-value and FC) of the 195 responsive genes in BY4741(*Δhap4*) and other deletion lines, with the values of h^2_{HAP4} also included.

Table S3: RNA-seq-based gene expression changes after deleting HAP2, HAP3, HAP4 and HAP5, respectively, in BY4741.

Table S4: Summary of the analyses of protein complexes in this study.

Table S5: Summary of the analyses of KEGG pathways in this study.

Table S6: The affected morphological traits in a variety of gene deletion lines.

Table S7: Summary of the clustered effects and distributed effects defined in each mutant that has public microarray data.

Table S8: Summary of the trait information of each diploid gene deletion or wild-type yeast strain, with the 405 trait values, the number of examined cells, and the number of replications included.

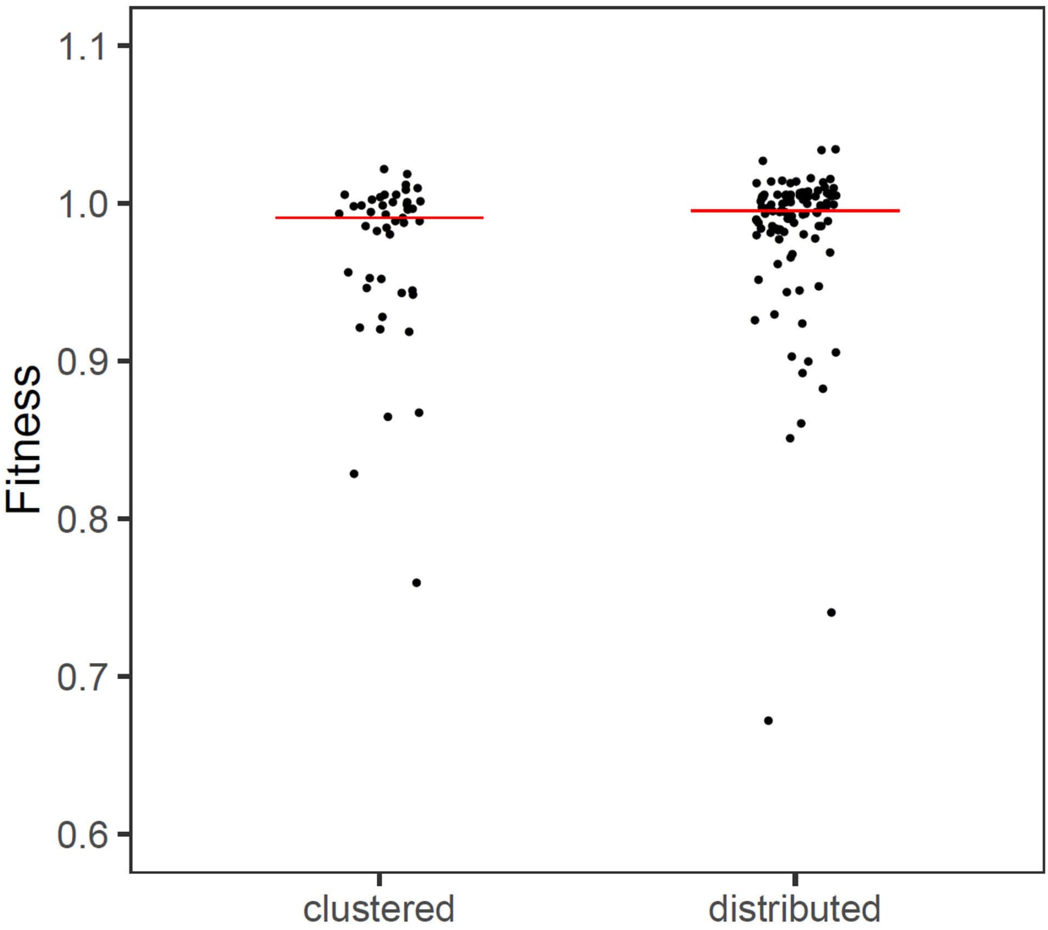

Fig. S1. Fitness importance is comparable between the clustered responsive genes and distributed responsive genes defined in BY4741(Δhap4) (*P* = 0.10, Mann-Whitney U-test). Fitness importance of a gene is measured by the relative growth rate of the gene deletion line to wild-type. The horizontal line shows the median.

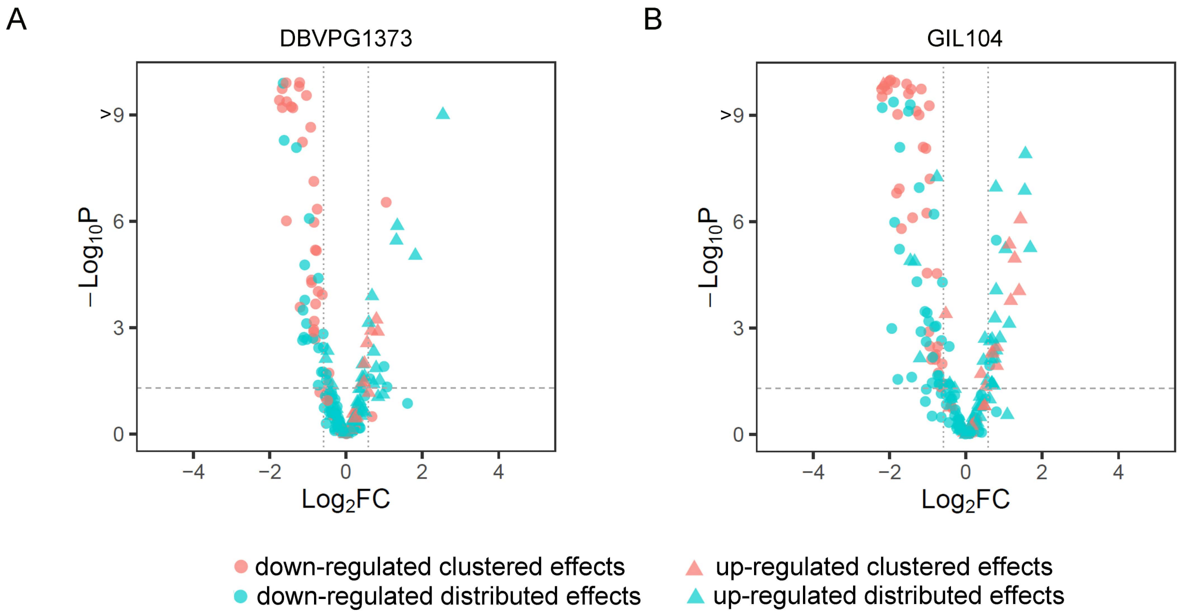

Fig. S2. The intra-species conservation analysis of the HAP4 deletion effects. The 195 responsive genes defined in BY4741(Δhap4) are examined with respect to their expression responses in *S. cerevisiae* DBVPG1373(Δhap4) and GIL104(Δ hap4), respectively. The horizontal dashed line shows adjusted P = 0.05 and vertical dashed lines show log2FC = ±0.58. (cyan: clustered effects; red: distributed effects; cycle: down-regulated in BY4741(Δhap4); triangle: up-regulated in BY4741(Δhap4)).

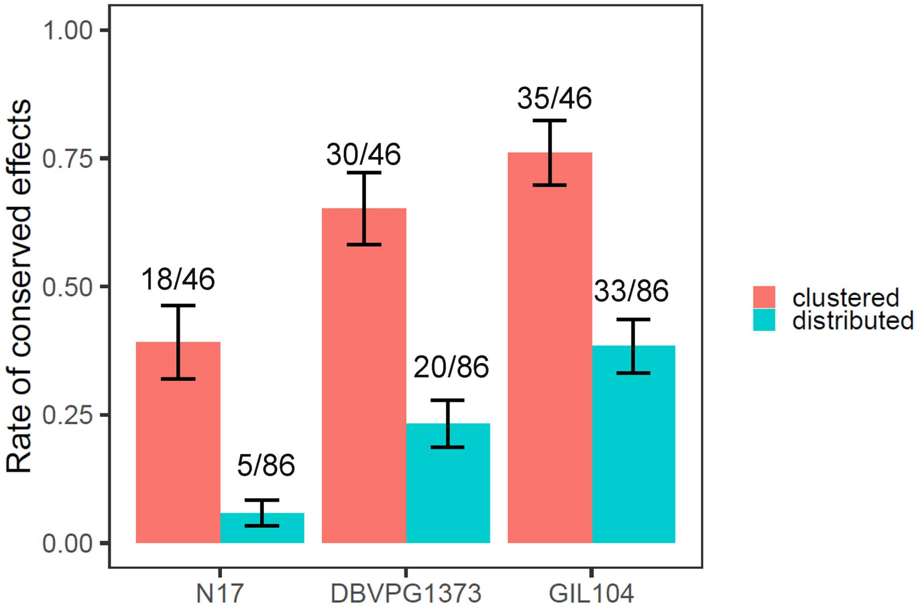

Fig. S3. Conservation analysis of the HAP4 deletion effects by considering only genes with a strong expression level in wild-type BY4741. This analysis is to address the concern that lowly expressed genes in wild-type tend not to have detectable down-regulation due to technical bias. Hence, for the 195 responsive genes defined in BY4741(Δhap4) only those with log2RPKM > 5 in wild-type BY4741 are considered here, leaving 46 clustered effects and 86 distributed effects. Error bars represent SE.

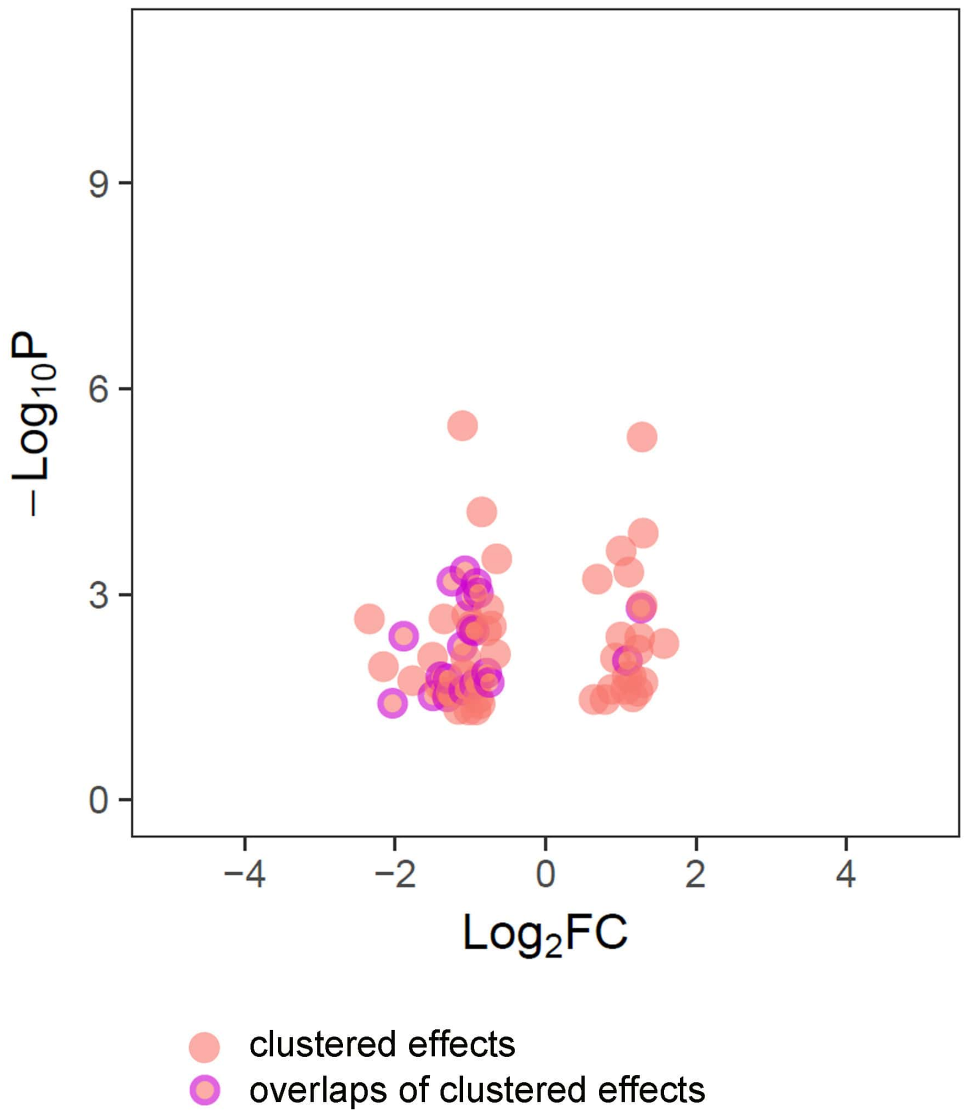

Fig. S4. The 20 overlapped clustered effects are comparable to the rest 45 (65-20) clustered effects defined in BY4741(Δhap4) with regard to their effect size. The differences are not statistically significant for both the P-values and fold changes observed in BY4741(Δhap4) (*P* = 0.75 and 0.51, respectively, Mann-Whitney U-test)

A

Deleted gene	Number of clustered effects	Number of distributed effects
HAP2	46	58
HAP3	140	316
HAP4	70	125
HAP5	53	108

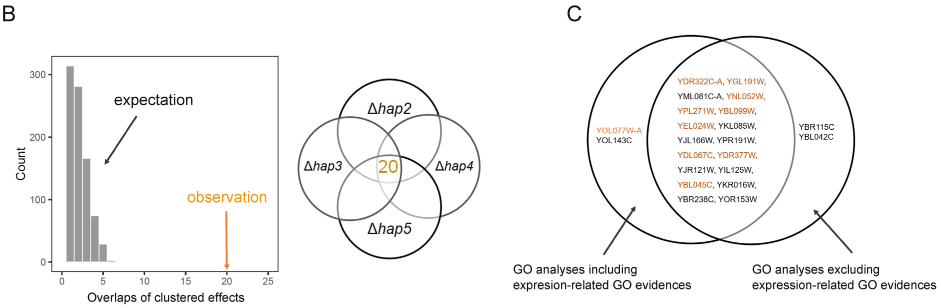

Fig. S5. The strong overlaps of clustered effects of the HAP2/3/4/5 complex genes are not biased by expression-related GO evidences for annotating the deletion effects. (A) Only the evidences IDA, HDA and IPI are used to re-define the clustered and distributed deletion effects of the four genes, respectively. (B) There are 20 overlapped clustered effects for the four genes encoding the HAP2/3/4/5 tetramer, which is significantly higher than expectation. The expectation is estimated by random sampling of the distributed effects of the four genes to calculate overlaps, and 1,000 such simulations were conducted. (C) The overlapped clustered effects are largely the same before and after excluding expression-related GO evidences. Genes that are the direct target of HAP4 are highlighted in yellow.

A

Deleted gene	Number of clustered effects	Number of distributed effects
HAP2	130	173
HAP3	33	41
HAP4	81	119
HAP5	12	39

B

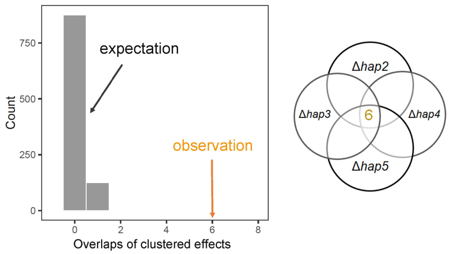

C

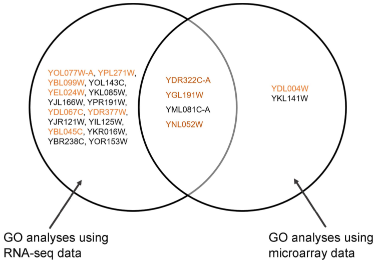

Fig. S6. The enrichment of overlapped clustered effects in HAP2/3/4/5 complex is reproduced by using public microarray data of the four gene deletion lines. (A) Microarray-based expression data are used to re-define the clustered and distributed deletion effects of the four genes, respectively. (B) There are only six overlapped clustered effects for the four genes encoding the HAP2/3/4/5 tetramer, which is significantly higher than expectation. The reduced number is primarily due to the small number (12) of clustered effects observed in HAP5 deletion. The expectation is estimated by random sampling of the distributed effects of the four genes to calculate overlaps, and 1,000 such simulations were conducted. (C) Comparison of the six overlapped clustered effects defined using microarray data with the 20 overlapped clustered effects defined using RNA-seq data. Genes that are the direct target of HAP4 are highlighted in yellow.

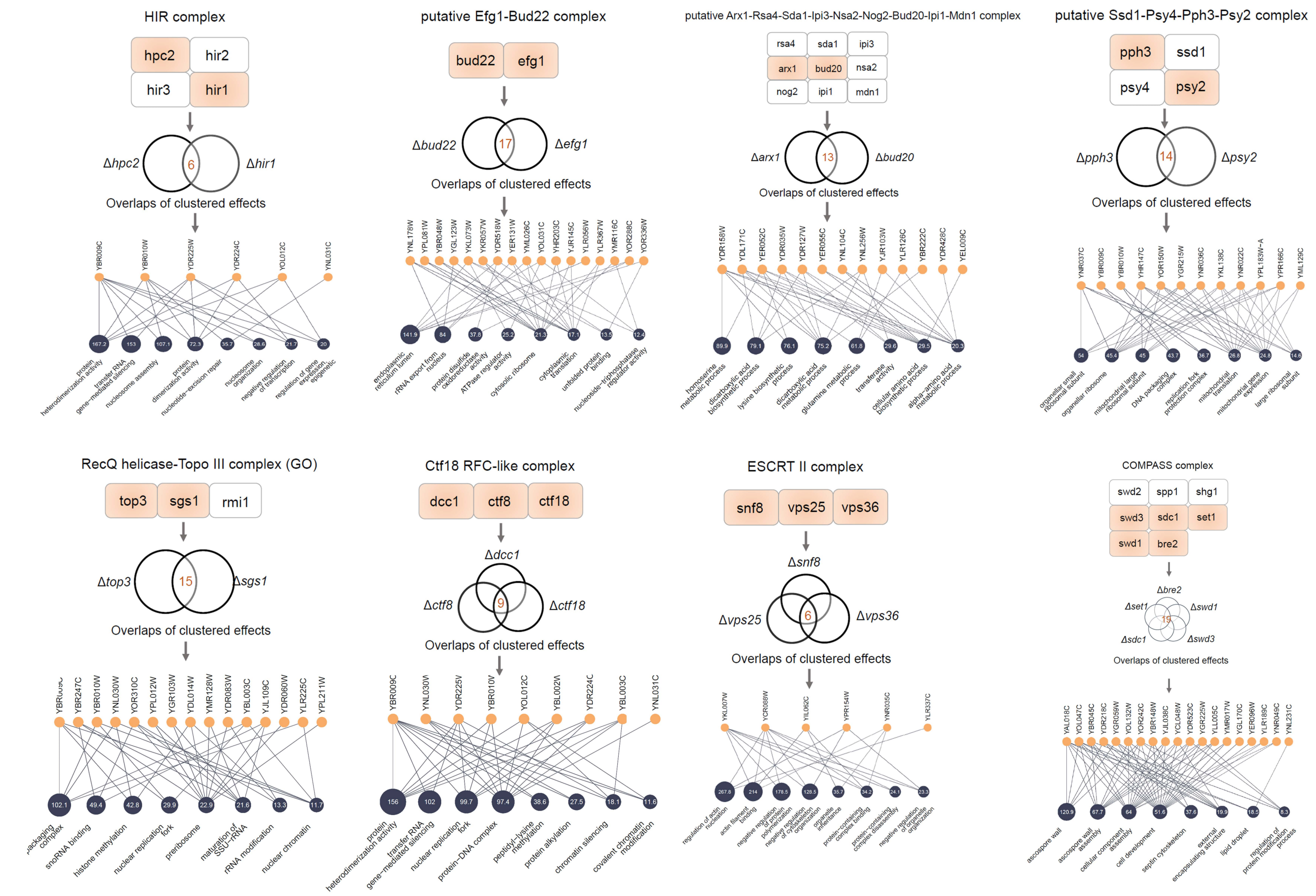

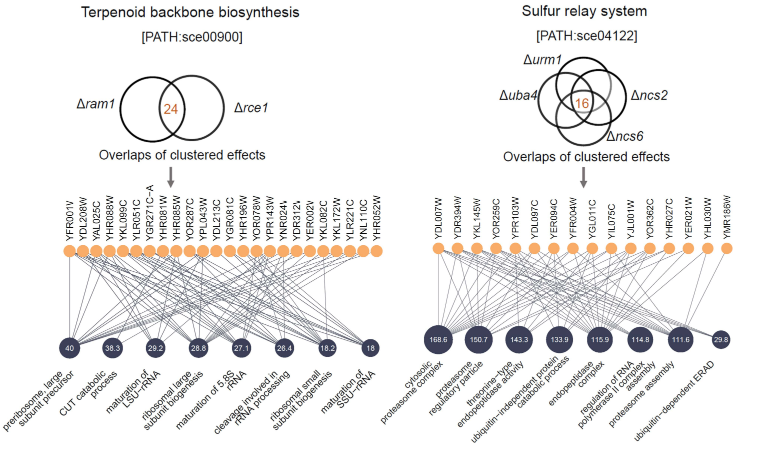

Fig. S7. Clustered effects in general support related biochemistry understandings much better than distributed effects. Ten protein complexes or KEGG pathways each with a moderate number (6-24) of overlapped clustered effects are shown. The enrichment rate of overlapped clustered effects relative to distributed effects for each of the eight protein complexes is 9.0, 5.7, 3.4, 10.4, 3.8, 29.4, 26.4, and over 100, respectively. For the two KEGG pathways the enrichment rate is 15.4 (PATH:sce00900) and 551.7 (PATH:sce04122), respectively. The member genes of each complex or pathway with suitable expression data for the analysis are highlighted in orange. The orange circle each represents an overlapped clustered effect, and the blue circles represent the enriched GO terms of the overlapped clustered effects with the number inside showing the fold enrichment in the given term.

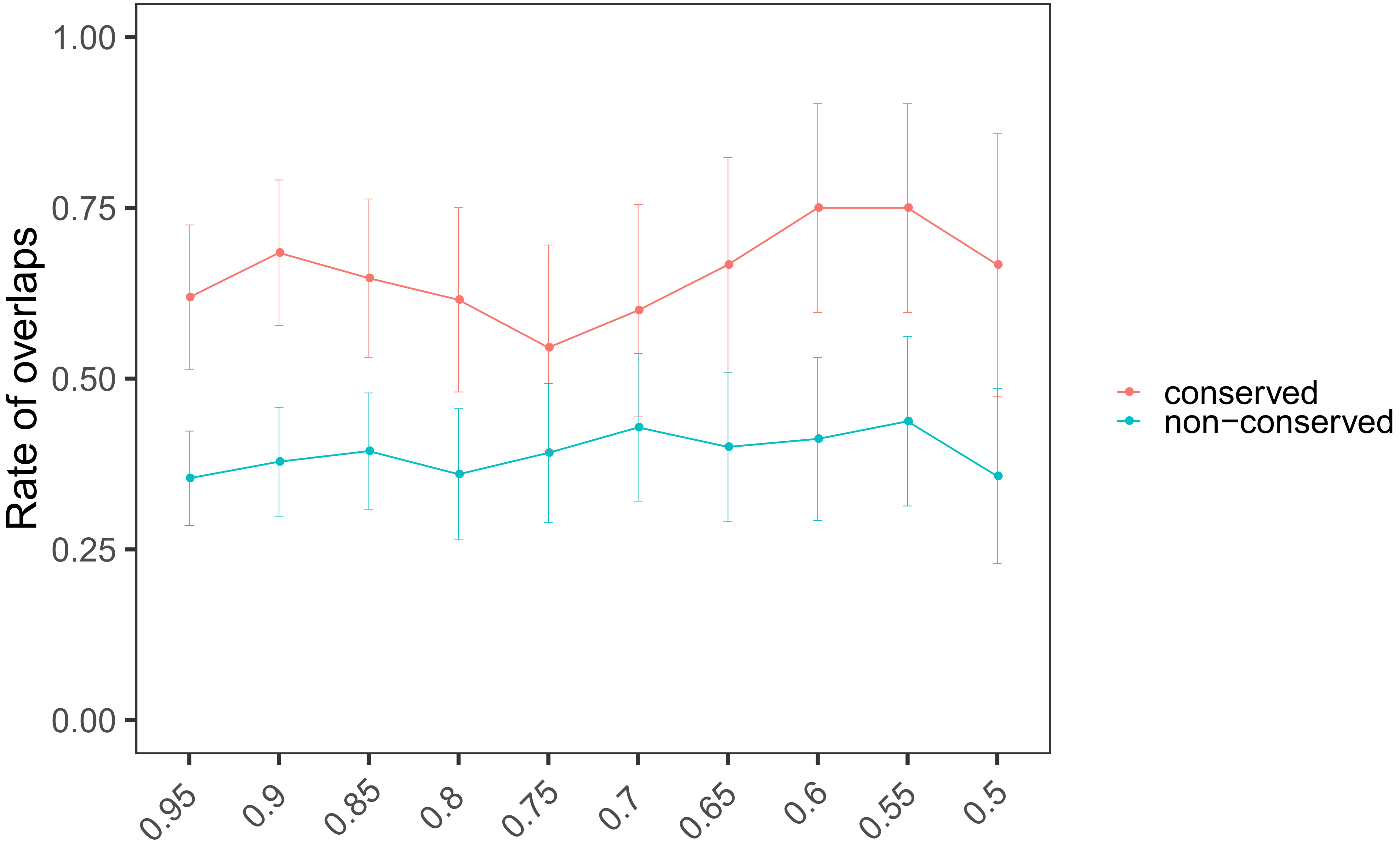

Fig. S8. The estimated rate of overlaps cannot be explained by correlated traits. The Pearson's R of all trait pairs is calculated using the trait values generated in ref. 4 for 4,718 yeast mutants. We then removed traits one by one from those with the highest absolute R until no two traits have R^2 greater than a threshold, which is set to be 0.95, 0.9, 0.85, 0.8, 0.75, 0.7, 0.65, 0.6, 0.55, and 0.5, respectively. The number of remaining traits are 346, 312, 277, 247, 223, 204, 185, 161, 153, and 127, respectively. Error bars represent SE.

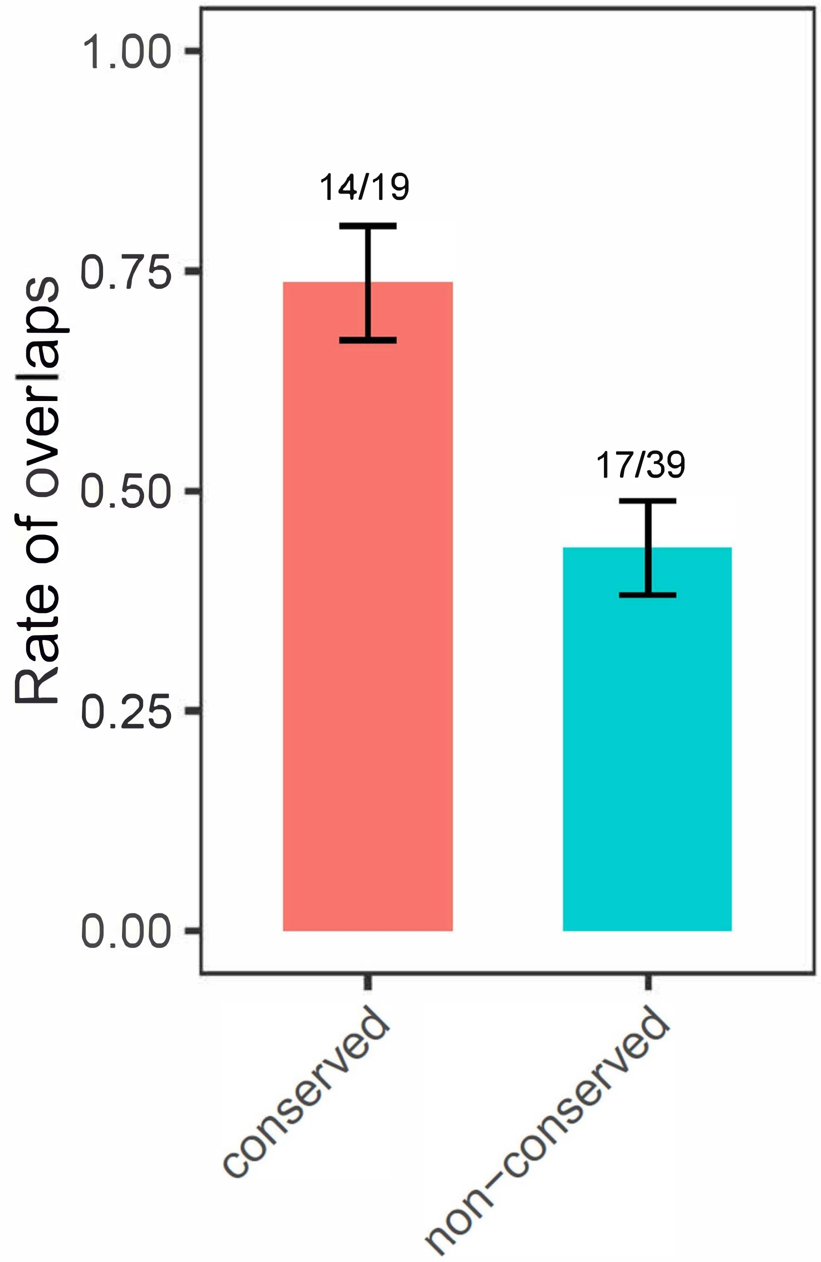

Fig. S9. The comparison in Fig. 4B is robust against trait measuring noise. To address the potential technical bias that traits with large measuring noise tend to be both non-conserved and non-overlapping, only traits with measuring CV < 0.1 across the replicates in wild-type BY4741 are considered. This results in 58 traits that are significantly affected by HAP4 deletion in S. cerevisiae, among which 19 are conserved effects and 39 non-conserved effects. The rate of overlaps in the conserved set remains significantly higher than the non-conserved set (P = 0.029, one-tailed Fisher's exact test). Overlaps refer to traits significantly affected by all four gene deletions in *S. cerevisiae*.

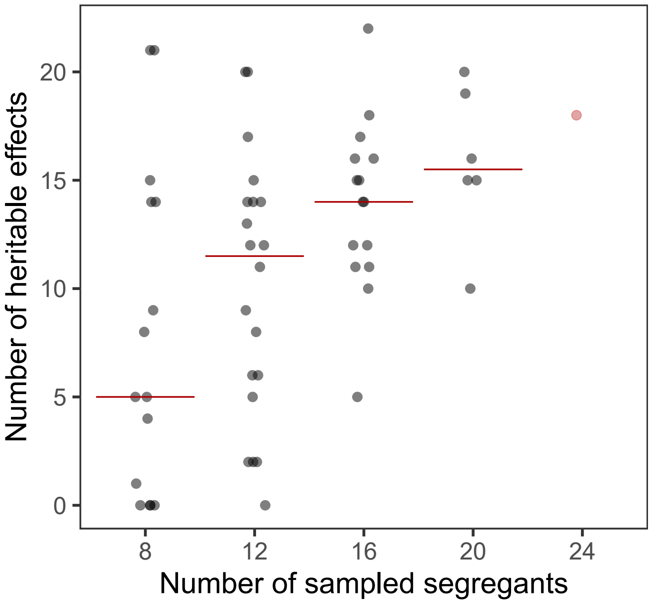

Fig. S10 The number of heritable effects obtained under 8(2 tetrads), 12(3 tetrads), 16(4 tetrads), or 20(5 tetrads) sub-sampled segregants. This analysis is to gauge how sample size of the segregant population affects the detection of heritable effects. Among the six tetrads examined in this study all combinations of 2, 3, 4, and 5 tetrads were considered, respectively. Each dot corresponds to a combination. The red line shows the median of the category.
